# The Regulation of 4-(methylnitrosamino)-1-(3-pyridyl)-1-butanone-Induced Lung Tumor Promotion by Estradiol in Female A/J Mice

**DOI:** 10.1371/journal.pone.0093152

**Published:** 2014-03-28

**Authors:** Rong-Jane Chen, Chu-Yung Chang, Louis W. Chang, Shih-He Siao, Yuan-Soon Ho, Chih-Hsiung Wu, Ning-Ping Foo, Pinpin Lin, Ying-Jan Wang

**Affiliations:** 1 Department of Environmental and Occupational Health, National Cheng Kung University Medical College, Tainan, Taiwan; 2 Graduate Institute of Clinical Medicine, Taipei Medical University, Taipei, Taiwan; 3 Visiting Chair Professor of Molecular Medicine, Institute of Molecular Medicine, National Cheng Kung University, Tainan, Taiwan; 4 Visiting Chair Professor, Toxicology Program, College of Pharmacy, Kaohsiung Medical University, Kaohsiung, Taiwan; 5 School of Medical Technology and Biotechnology, Taipei Medical University, Taipei, Taiwan; 6 Department of Surgery, School of Medicine, Taipei Medical University-Shuang Ho Hospital, Taipei, Taiwan; 7 Center of Excellence for Cancer Research, Taipei Medical University, Taipei, Taiwan; 8 Department of Emergency Medicine, Chi-Mei Medical Center, Liouying, Tainan, Taiwan; 9 Department of Emergency Medicine, Tainan Municipal An-Nan Hospital, China Medical University, Tainan, Taiwan; 10 Division of Environmental Health and Occupational Medicine, National Health Research Institutes, Zhunan Town, Taiwan; Taipei Medicine University, Taiwan

## Abstract

Epidemiological studies indicate that women are at a higher risk developing lung cancer than men are. It is suggested that estrogen is one of the most important factors in lung cancer development in females. Additionally, cigarette smoke, and environmental pollutants, such as 2,3,7,8-tetrachlorodibenzo-p-dioxin (TCDD), may play salient roles in female lung carcinogenesis. However, the mechanisms responsible for the interaction of these factors in the promotion of lung cancer are still poorly understood. The present study was designed to explore two ideas: first, the synergistic lung tumorigenic effects of 4-(methylnitrosamino)-1-(3-pyridyl)-butanol (NNK) combined with TCDD, 17β-estradiol (E2) or both through a long-term treatment experiment, and second, to identify early changes in the inflammatory and signaling pathways through short-term treatment experiments. The results indicate that A/J mice given E2 had strong effects in potentiating NNK-induced activation of MAPK signaling, NFκB, and COX-2 expression. In the long-term exposure model, E2 had a strong tumor promoting effect, whereas TCDD antagonized this effect in A/J mice. We conclude that treatment with NNK combined with either E2 or TCDD induces lung carcinogenesis and the promotion effects could be correlated with lung inflammation. E2 was shown to potentiate NNK-induced inflammation, cell proliferation, thereby leading to lung tumorigenesis.

## Introduction

The tobacco-specific compound nitrosamine 4-(methylnitrosamino)-1-(3-pyridyl)-butanol (NNK) is the most powerful tobacco carcinogen [1]. NNK and its metabolites induce lung cancer and cancer cell growth through genotoxic and epigenetic mechanisms. Among these mechanisms, NFκB is reported to be the key mediator of NNK exposure in the development of lung cancer [2]. Through activation of NFκB-related pathways, NNK increases the release of inflammatory cytokines including TGF-β, IL-1, IL-8, and G-CSF [3]. These inflammatory mediators may further promote neoplasia by inducing preneoplastic mutation, proliferation, resistance to apoptosis, invasiveness, angiogenesis, and secretion of immune suppressive factors [3]. Aside from the tumorigenic effects of NNK alone, emerging data suggested that other environmental toxicants are also considered to play significant roles in the development of lung cancer [4]. 2,3,7,8-tetrachlorodibenzo-p-dioxin (TCDD) is a member of the polyhalogenated aromatic hydrocarbon family and is one of the most toxic environmental compounds produced by industrial combustion and chemical manufacturing processes [5]. We have recently reported that the relationship between TCDD exposure and cigarette smoking could be synergistic in the development of lung cancer in female A/J mice [6].

Lung cancer is the leading cause of cancer deaths worldwide in men and women. Exposure to tobacco smoke remains the most prominent risk factor for lung cancer development, with 90% of female lung cancer deaths being attributed to smoking [7]. However, independent of tobacco exposure, women are at greater risk of lung cancer than men [5]. Epidemiological and clinical data also suggest a gender difference in the etiology of lung cancer [8]. Therefore, additional lung cancer risk factors in females should be taken into consideration [9]. Estrogen is a recognized factor in the pathogenesis of breast, endometrial, and ovarian cancers [10]. Fu *et al.* reported that significantly more women under 50 years old were diagnosed with lung cancer when they are largely premenopausal, than men who were younger than 50 years old [11]. These observations suggest that estrogen could be an important factor in lung carcinogenesis [10].

Strong correlation between estrogen and lung cancer development have been reported before. For example, estrogen governs many physiological functions including cell growth, differentiation, and development through estrogen receptor (ER)-mediated signaling pathways in cancer cells [12], [13]. Upon activation by estrogen, ER causes the proliferation of lung cancer cells through the activation of several growth factor genes, such as tumor necrosis factor-α (TNF-α), epidermal growth factor (EGF), and insulin-like growth factor-1 (IGF-1) that are known to mediate cell division in lung neoplasia [14]. 17β-estradiol (E2) and aromatase, the enzyme that converts testosterones to E2 are elevated in lung cancer tissues [15]. In addition to the ER-dependent activity, the estrogen metabolites such as OHE2 increased the NFκB and COX-2 expression in lung cells [16]. Therefore, female mice exposed to cigarette smoke showed more vigorous inflammatory reactions and generated more toxic oxidative stress in their airways than did male [17].

Accordingly, these findings suggest that cigarette smoke, estrogen, and other environmental pollutants play salient roles in female lung carcinogenesis that is associated with chronic inflammation. Hence, we intend to investigate the possible influence of TCDD, 17β-estradiol (E2), or both on the regulation of NNK-induced lung carcinogenesis in A/J mice that has been shown to be the strain most sensitive to cigarette smoke exposure [18]. The present study was designed to explore the lung tumorigenic potential of NNK combined with TCDD, E2 or both through a long-term treatment experiment and the possible contribution of inflammatory signaling pathways in lung tumorigenesis through a short-term treatment experiment.

## Materials and Methods

### 2.1 Chemicals and reagents

17β-estradiol (E2) was purchased from Sigma Aldrich (Saint Louis, MO, USA). TCDD and NNK were obtained from Toronto Research Chemicals Inc. (TRC, North York, Ontario, Canada). E2 was dissolved in corn oil, and TCDD was dissolved in dimethylsulfoxide (DMSO) and stored in the dark at −20°C until use. NNK was dissolved in normal saline solution immediately before use. Primary antibodies against IκB, phospho-IκB, NFκB subunit p65, p50, COX-2, cyclin D1, ERK1/2, phospho-ERK1/2, JNK, phospho-JNK, p38, phospho-p38, PCNA, iNOS, MMP-9, GAPDH, and horseradish peroxidase (HRP)-conjugated anti-mouse and anti-rabbit secondary antibodies were purchased from Cell Signaling (Beverly, MA, USA).

### 2.2 Surgery, chemical treatments, and tissue preparations

**Figure 1:**
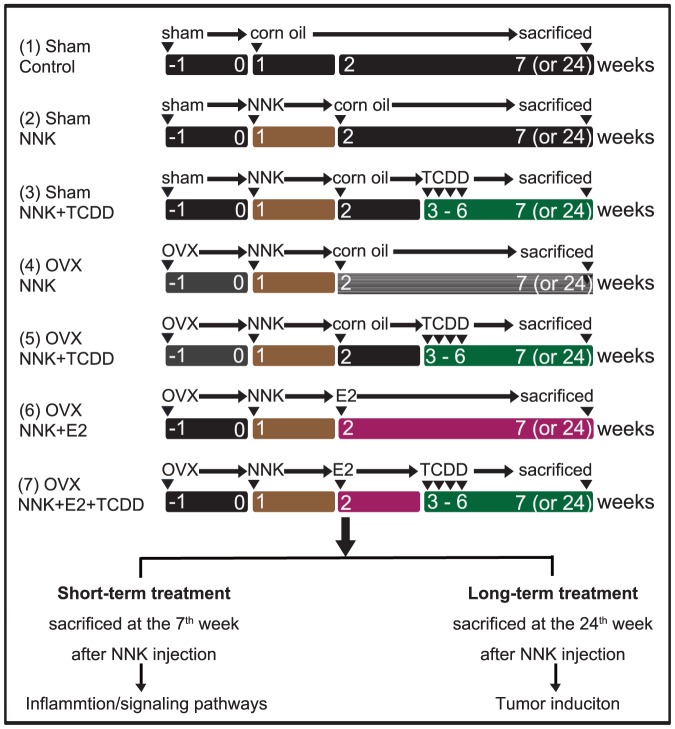
Experimental design. Female A/J mice were divided into 7 groups in which groups 1–3 were sham-operated (Sham) and groups 4–7 were ovariectomized (OVX). Mice were OVX 2 weeks before given NNK. Group 1 is the sham-operated control group in which mice were given corn oil i.p. 3 times per week until the end of the experiment. Groups 2–7 were pretreated with NNK (1 mg/0.1 ml/mouse, i.p.) at the 1^st^ week of the experiment. Groups 3, 5, and 7 received a loading a dose of TCDD (5 µg kg/b.w./i.p.) from week 3 and the maintaining dose of TCDD (1.42 µg kg/b.w./i.p) 3 times weekly from week 4 for 3 weeks. In week 2, groups 6 and 7 were exposed to 17β-estradiol (E2, 2 µg/kg/b.w.) subcutaneously every 2 days continuously until the end of the experiments. The experiments were terminated after the 7^th^ week as a short-term exposure model to detect the inflammatory response, and terminated at the 24^th^ week as a long term exposure model to evaluate lung tumor formation.

Six-week old female A/J mice, acquired from the Laboratory Animal Center of the National Cheng Kung University Medical College, were housed (five mice per cage) in a pathogen-free environment, maintained on Lab Diet 5010 (PMI Feed, Inc, USA) at 24±2°C and, 50%±10% relative humidity, and were subjected to a 12-h light/12-h dark cycle. Animal studies were approved by the Laboratory Animal Center of the National Cheng Kung University Medical College and performed according to the local guidelines for animal care and protection (Permit number: 98112). The experimental design for the treatment groups and the duration of exposure is shown in Figure 1.

At 7 weeks of age, mice were sham operated (Sham) or ovariectomized (OVX) by bilateral dorsal incision under anesthesia (0.15 ml/0.02 kg b.w. of a 1∶1 solution of hypnorm and dormicum diluted a further 1∶1 in sterile water s.c.). Analgesia (0.1 ml 10% buprenorphine/0.02 kg b.w.) was given during the recovery from surgery. The surgically treated animals were housed singly and given 2 weeks to recover. The exposure experiments were performed out at 9 weeks of age for all animals. The mice were randomly divided into 7 groups, in which groups 1–3 were the sham-operated mice, and groups 4–7 were OVX mice. Treatment of the 7 groups was as follows: (1) Sham Control group, i.p. corn oil 0.1 ml every two days until the end of the experiment; (2) Sham NNK group, a single i.p. injection of NNK followed by i.p. corn oil 0.1 ml every two days until the end of the experiment; (3) Sham NNK+TCDD group, a single injection of NNK followed by given 0.1 ml of corn oil every two days for 1 week. At the 3^rd^ week, mice were given a loading dose of TCDD followed by the weekly maintenance doses of TCDD for 3 weeks; (4) OVX, NNK group, a single i.p. injection of NNK followed by i.p. corn oil 0.1 ml every two days until the end of the experiment; (5) OVX, NNK+TCDD group, mice were given a single dose of NNK at the first week, followed by giving corn oil for 1 week. Mice were then given a loading dose of TCDD followed by the weekly maintenance doses of TCDD for 3 weeks; (6) OVX, NNK+E2 group, mice were given a single dose of NNK then given E2 subcutaneously from the 2^nd^ week until the end of the experiments; and (7) OVX, NNK+E2+TCDD group, mice were given a single dose of NNK followed by E2 at the 2^nd^ week until the end of the experiments. At the 3^rd^ week, mice were given a loading dose of TCDD followed by the weekly maintenance doses of TCDD for 3 continuous weeks.

NNK (1 mg/0.1 ml/mouse) was given intraperitoneally at the 1^st^ week of the experiment (Group 2–7). At the 2^nd^ week, E2 (2 µg/kg/b.w.) was given subcutaneously every 2 days until the end of the experiment (Group 6, 7). The loading dose of TCDD (5 µg kg/b.w./i.p.) was given at the 3^rd^ week of the experiment, then the maintenance dose of TCDD (1.42 µg/kg/b.w./i.p.) was given 3 times a week for an additional 3 weeks (Group 3, 5, 7). The doses of TCDD were modified from those described in published studies, which showed successful enhancement of lung tumorigenesis in mice [6]. To delineate the effects of NNK combined with E2, and/or TCDD in the inflammatory response, we designed a short-term exposure experiment (terminated at the 7^th^ week after NNK treatment) and a long-term experiment (terminated at the 24^th^ week after NNK treatment) to evaluate lung tumor induction. At the end of the experiments, all mice were euthanized under ether anesthesia, and serum was obtained from cardiac blood. Lung tissues were excised, weighed, and examined for the presence of gross tumors at necropsy and were then sectioned and fixed in 10% paraformaldehyde or frozen in liquid nitrogen.

### 2.3 Histology and immunohistochemistry (IHC)

Lung tissue sections (4 µm) were sliced from paraffin-embedded, formalin-fixed lungs and stained with hematoxylin and eosin (H&E). For immunohistochemistry, tissue sections were deparaffinized and hydrated. Antigen retrieval was performed in a microwave for 20 min with citrate buffer (pH 6.0), and then the slides were immersed in 3% H_2_O_2_ for 20 min to block the activity of endogenous peroxidase. After blocking, the slides were incubated overnight at 4°C using primary antibodies at a 1∶100 dilution and then incubated with the appropriate secondary antibody for 1 hr at room temperature. The slides were developed with the STARRTREK Universal HRP detection Kit (Biocare medical, Concord, CA, USA) according to the manufacturer's protocol, and the slides were counterstained with hematoxylin.

### 2.4 Determination of the TGF-β and TNF-α Levels

Mouse serum was used to determine the levels of TGF-β and TNF-α using an ELISA kit according to the manufacturer's instructions (R&D systems, Inc, Minneapolis, MN, USA).

### 2.5 Western blot analyses

Randomized frozen lung samples from 3 individual mice from different groups were homogenized, and the lysates were subjected to gel electrophoresis and immunoblotting. Cytoplasmic extracts and nuclear extracts were performed by using Buffer A (10 mM HEPES, pH 7.8, 10 mM KCl, 2 mM MgCl_2_, 1 mM DTT, 0.1 mM EDTA, 0.1 mM PMSF), Buffer B (500 µl buffer A containing 0.5% NP-40), and Buffer C (50 mM HEPES, PH7.8, 50 mM KCl, 300 mM NaCl, 1 mM DTT, 0.1 mM EDTA, 0.1 mM PMSF, 20 µl glycerol). Briefly, lung tissues were homogenized with Buffer A. Following centrifugation, the supernatant were collected as cytoplasmic extracts, and the pellet were washed with Buffer B then re-suspended with Buffer C. Following extraction, the nuclei were removed by centrifugation, and the supernatants were collected as the nuclear extracts. Immunoreactive proteins were visualized with a chemiluminescent detection system (PerkinElmer Life Science, Inc. MA, USA) and BioMax LightFilm (Eastman Kodak Co., New Haven, CT, USA) according to the manufacturer's instructions.

### 2.6 Statistical analyses

The results are expressed as the mean ± standard error of the mean (SEM). The experimental data were analyzed using Student's *t*-test. Data of tumor multiplicity were analyzed by Fisher exact test. Differences were considered to be statistically significant when the p value was less than 0.05.

## Results

### 3.1 General health status of the short-term and long-term treatment groups

To test the role of inflammation associated with lung tumorigenesis, sham-operated or ovariectomized A/J mice were exposed to NNK alone, NNK combined with E2, TCDD or both, and the experiment was terminated at the 7^th^ week after NNK injection (short-term experiment, Figure 1). The effects of NNK combined with TCDD, E2, or both on lung tumor promotion were examined in the long-term experiment, in which A/J mice were sacrificed at the 24^th^ week after NNK injection (Figure 1). The changes in body weight and lung weight are summarized in Table 1. The mean body weights (BW) and lung weights (LW) increased with time among all groups, but the LW/BW ratio did not display significant changes at any point through the experimental period (Table 1).

**Table 1:** Average lung and body weights of the mice in the short-term (7 wks) and long-term (24 wks) exposure models.

NNK, E2, and TCDD effects on lung and body weight increases after treatment for 7 or 24 weeks								
Treatment	Mice numbers		Body weight (BW, g)		Lung weight (LW, g)		LW/BW ration (%)	
	7 wks	24 wks	7 wks	24 wks	7 wks	24 wks	7 wks	24 wks
Sham, Control	4	36	16.7±2.06	22.7±2.94	0.11±0.01	0.14±0.02	0.68±0.08	0.61±0.07
Sham, NNK	4	36	16.8±2.63	22.8±2.18	0.09±0.01	0.13±0.02	0.59±0.09	0.59±0.10
Sham, NNK+TCDD	4	26	14.8±2.71	23.2±2.1	0.12±0.01	0.14±0.02	0.83±0.13	0.60±0.08
OVX, NNK	4	18	18.3±1.26	25.2±2.49	0.10±0.01	0.14±0.03	0.60±0.05	0.55±0.08
OVX, NNK+TCDD	4	18	19.0±1.63	25.2±1.74	0.10±0.01	0.13±0.01	0.55±0.09	0.53±0.06
OVX, NNK+E2	4	18	20.2±2.16	24.2±1.93	0.11±0.02	0.13±0.02	0.56±0.06	0.53±0.08
OVX, NNK+E2+TCDD	4	20	18.6±1.95	25.3±2.54	0.12±0.01	0.12±0.02	0.66±0.06	0.49±0.05

Sham: sham-operated, OVX: ovariectomized, LW: lung weight, BW: body weight. E2: 17β-estradiol. Data are presented as the mean ± SEM.

### 3.2 Tumor-Promoting effects of E2, TCDD and both on NNK-induced lung adenoma formation

**Figure 2:**
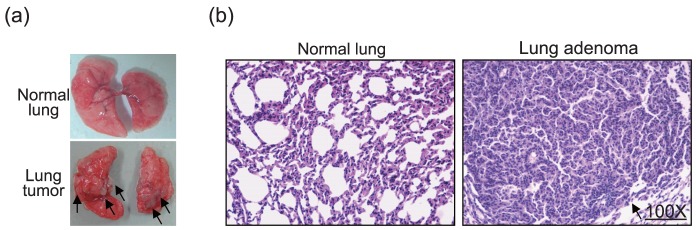
Examination of lung tumors on A/J mice. Tumor pathology of lung tumors in A/J mice treated with NNK for 24 weeks. (a) Images of the lung surface indicate that NNK-treated mice lungs presented with numerous visible lesions (black circles) and (b) the histological characteristics of mouse lungs stained with H&E from control and NNK treatment groups. Arrows indicate the adenocarcinoma in the lungs (magnification, ×100).

The lung adenoma formation observed in the long-term experiments showed that lung tumors with white coloration were located in the subpleural area (Figure 2a, sub panel). The lung tumors were composed of cuboid cells with hyperchromatic nuclei. This histological feature was consistent with adenoma (Figure 2b, right panel). The tumor incidence and multiplicity of control of each treatment group are listed in Table 2. The sham-operated control mice showed a 5.6% occurrence of spontaneous lung adenomas (group 1), whereas treatment with NNK (group 2) significantly increased tumor incidence (19.4%) and multiplicity (0.17±0.04), suggesting that NNK significantly induced lung tumor formation in female mice. Combination treatment with NNK and TCDD in the sham-operated A/J mice significantly increased tumor incidence (34.6%) and multiplicity (0.25±0.03) compared to the control group (group 3 vs. group 1). Similar results were also observed in the OVX mice received NNK combined with TCDD (group 5) that tumor incidence (33.3%) and multiplicity (0.38±0.17) were higher than single NNK injected in OVX mice (group 4). The results confirmed that TCDD enhances lung tumorigenesis induced by NNK; however, the role of E2 in the lung tumorigenesis did not clarify among these groups.

**Table 2:** The incidence and lung tumor multiplicity of lung tumor formation in A/J mice treated with NNK, NNK combined with TCDD, E2, or both.

The prevalence and tumor multiplicity of lung tumor formation in female A/J mice				
Treatment	Mice numbers	Tumor bearing mice	Prevalence (%)	Tumor multiplicity
Sham, Control	36	2	5.6	0.07±0.02
Sham, NNK	36	7	19.4	0.17±0.04*
Sham, NNK+TCDD	26	9	34.6	0.25±0.03*
OVX, NNK	18	2	11.1	0.17±0.08
OVX, NNK+TCDD	18	6	33.3	0.38±0.17* ^,^ §
OVX, NNK+E2	18	10	55.6	2.06±0.24* ^,^ † ^,^ # ^,^ ‡
OVX, NNK+TCDD+E2	20	20	50	0.55±0.04* ^,^ # ^,^ §

* Significantly higher (*p*<0.05) compared to Sham, control groups.

† Significantly higher (*p*<0.05) compared to Sham, NNK groups.

# Significantly higher (*p*<0.05) compared to OVX, NNK groups.

‡ Significantly higher (*p*<0.05) compared to OVX, NNK+TCDD groups.

§ Significantly higher (*p*<0.05) compared to OVX, NNK+E2 groups.

Tumor incidences are given as the number of animals with tumors/total number of animals at risk (%). Tumor multiplicity is the average tumor number on tumor-bearing mice. Data of lung tumor multiplicity are given as the mean ± SEM.

*significantly higher (*p*<0.05) compared to Sham, Control groups,

# significantly higher (p<0.05) compared to OVX, NNK groups,

† significantly higher (*p*<0.05) compared to Sham, NNK groups,

‡ significantly higher (*p*<0.05) compared to OVX, NNK+TCDD groups,

§ significantly higher (*p*<0.05) compared to OVX, NNK+E2 groups.

To explore the role of E2 in potentiating lung tumorigenesis, the E2-attributable effects on NNK-induced lung tumorigenesis were analyzed. OVX mice treated with NNK (group 4) showed lower tumor incidence compared to sham NNK group (group 2), suggested that female hormone may regulated lung tumorigenesis. Addition of E2 combined with NNK in the OVX mice (group 6) showed the highest tumor incidence (55.6%) and multiplicity (2.06±0.24) among all groups following 24 weeks of exposure (Table 2). The results suggested that E2 has a strong enhancing effect on NNK-induced lung tumors.

Compared with the effect of TCDD and E2 in NNK-induced lung tumor, the OVX mice supplemented with E2 (group 6) showed significantly higher tumor multiplicity (2.06±0.24) than OVX mice treated with NNK+TCDD (group 5, 0.38±0.17) indicating that tumor promoting effect was significant in the E2+NNK group (group 6) than TCDD+NNK group (group 5). However, simultaneous administration of E2 and TCDD to NNK-treated mice (group 7) resulted in significantly decreased tumor incidence (50%) and multiplicity (0.55±0.04) compared to the NNK+E2 group (group 6) (Table 2). The results indicated that E2 possesses greater tumor-promoting effects than TCDD; TCDD did not potentiate but somehow antagonized the E2 tumor-promoting effects in A/J mice (Table 2).

### 3.3 The effect of interactions between NNK and E2, NNK and TCDD on the inflammatory response in a short-term experiment

**Figure 3:**
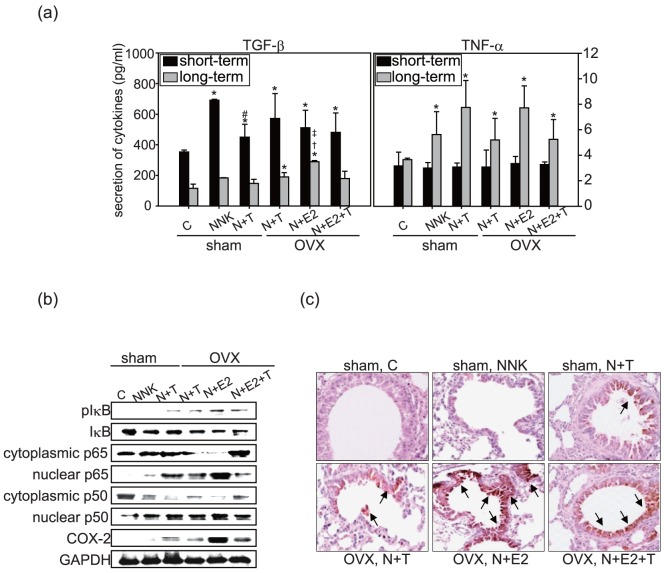
Inflammatory responses in A/J mice. (a) TGF-β (left panel) and TNF-α (right panel) expression in serum by treatment groups. The results are expressed the means ± SEM (n = 3 in each group). *p<0.05 compared to sham control groups. ^#^ *p*<0.05 compared to short-term sham NNK groups. ^†^ *p*<0.05 compared to long-term OVX, N+T groups. ^‡^ *p*<0.05 compared to long-term OVX, N+E2+T groups. Short term: experiment terminated at the 7^th^ week. Long term: experiment terminated at the 24^th^ week. (b) Lung homogenates were used to detect the expression and activation of pIκB, IκB, cytoplasmic p65, nuclear p65, cytoplasmic p50, nuclear p50, COX-2 and GAPDH by using specific primary antibodies. (c) Immunohistochemistry for COX-2 protein in A/J mice lung tissues. Arrows indicated COX-2 positive stained bronchial epithelium (magnification, ×100). Sham: sham-operated, OVX: ovariectomized, C: control, N+T: NNK+TCDD, N+E2: NNK+E2 (17β-estradiol), N+E2+T: NNK+E2+TCDD.

Based on the previous reports, which showed a link between lung inflammation and carcinogenesis [19], we evaluated the expression of inflammatory cytokines using mouse serum. Our results indicated that tumor multiplicity of OVX NNK group (group 4) was similar to sham NNK group (group 2), therefore, mouse serum and lung tissues selected from group 1–3 (sham-operated groups), and group 5–7 (OVX groups) were used for further analysis. OVX NNK group (group 4) was excluded in the following experiments. As shown in Figure 3, compared with the sham control group, TGF-β levels increased in the sham-operated mice receiving NNK, NNK+TCDD, or OVX mice receiving NNK+TCDD, NNK+E2, and NNK+E2+TCDD in the short-term experimental study (Figure 3a, left panel). OVX mice exposed to E2 did not show differences in TGF-β levels compared to the other OVX groups (Figure 3a, left panel). The results indicate that NNK combined with TCDD, E2, or both had similar effects on the induction of TGF-β levels. Interestingly, in the long-term treatment groups, the levels of TGF-β were not significantly changed, except for the NNK+E2 groups, suggesting that E2 continuously stimulates the production of TGF-β. In contrast, TNF-α levels were significantly elevated only in the sham and OVX mice receiving NNK, NNK combined with TCDD, E2 or both in long-term treatment groups (Figure 3a, right panel). The results implied a different expression pattern of TGF-β and TNF-α, showing that TGF-β was affected by the treatment in the short-term experiment whereas the expression of TNF-α is increased after long term treatment.

We then determined whether administration of E2, TCDD or both affected NNK's ability to induce an inflammatory response in lung tissues using COX-2 expression and the NFκB pathways as inflammatory markers. The results showed that sham and OVX mice exposed to NNK+TCDD, the expression of phospho-IκB, nuclear p65, nuclear p50, and IκB degradation were all significantly increased compared to sham NNK group (Figure 3b). In OVX mice treated with NNK+E2 or NNK+E2+TCDD, the expression of phospho-IκB, nuclear p65, nuclear p50, COX-2, and IκB degradation were also significantly increased while the expression of cytoplasmic p65 and p50 were decreased. The most significant increases in expression of the nuclear NFκB subunits p65 and p50, as well as COX-2 were found in the NNK+E2 groups (Figure 3b). The immunohistochemistry results of the short-term experimental groups for COX-2 expression further demonstrated that the distribution of COX-2 positive stained bronchial epithelial cells in OVX mice treated with NNK+E2 was more widespread than in other treatment groups (Figure 3c). These results suggested that the pro-tumorigenic effects of NNK and the potentiated tumorigenic effects when combined with TCDD or E2 are convergent on a persistent/dysregulated inflammatory response. Nevertheless, co-administration of NNK+E2+TCDD induced less COX-2 expression than the NNK+E2 group, implying an antagonistic response could have occurred between TCDD and E2.

### 3.4 Alteration of MAPK signaling pathways

**Figure 4:**
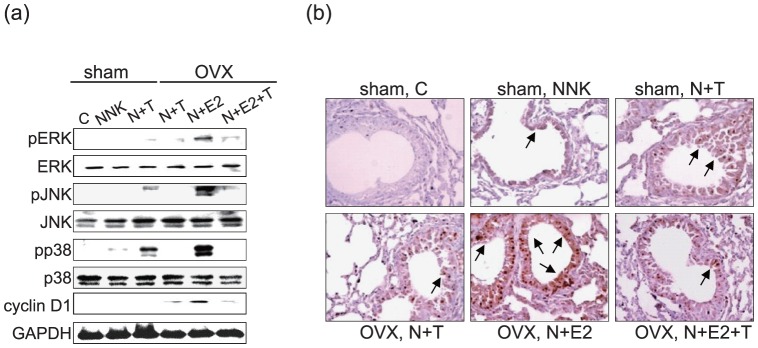
The involvement of the MAPK cascades and the PCNA expression in short-term exposure A/J mice. (a) Lung homogenates were collected from each group of A/J mice. Expression and phosphorylation of pERK, ERK, pJNK, JNK, pp38, p38, cyclin D1, and GAPDH were analyzed by western blot analysis. (b) Immunohistochemistry was used to detect the expression of PCNA (cell proliferation marker) in lung tissues. The arrows indicated positive staining for PCNA in bronchial epithelium (magnification, ×100). Sham: sham-operated, OVX: ovariectomized, C: control, N+T: NNK+TCDD, N+E2: NNK+E2 (17β-estradiol), N+E2+T: NNK+E2+TCDD.

It has been reported that NNK promotes the activation of MAPK pathways in cancer cells [1]. E2 can also activate MAPK pathways, leading to the proliferative and anti-apoptotic effects observed in a variety of cell types [20]. We hypothesized that the interaction between NNK and E2 in promoting inflammation may be through the co-activation of MAPK signaling pathways. Three major MAPK signaling pathways were detected by western blot analyses, and the results revealed that ERK1/2, p38, and JNK were significantly phosphorylated and activated upon challenged with NNK combined E2 (Figure 4a). Moreover, we also revealed that the combined treatment induced overexpression of cyclin D1, which is a key regulator of cell cycle progression (Figure 4a). The immunohistochemistry results further confirmed that NNK combined with E2 induced potent overexpression of the cellular proliferation as determined by the proliferative marker PCNA in the bronchial epithelium (Figure 4b). Nevertheless, NNK combined with TCDD also induced activation of MAPK pathways, cyclin D1 and PCNA expression but to a lesser extent (Figure 4a and 4b). The results indicated that NNK combined with TCDD or E2 induces lung tissue inflammation and cellular proliferation and may require the activation of MAPK signaling pathways. NNK combined with E2 was more prone to activate MAPK pathways, cyclin D1 and PCNA.

### 3.5 Alteration of cancer cell proliferation, inflammation, and progression

**Figure 5:**
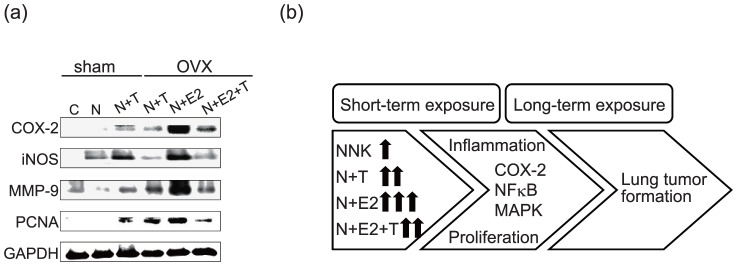
The inflammatory and proliferative protein expression pattern in the long-term exposure groups. (a) Lung homogenates were collected and analyzed for the expression of the inflammatory markers COX-2 and iNOS, proliferative marker PCNA, and MMP-9 by western blot analysis. (b) A model for the synergistic effects of NNK+E2, or NNK+TCDD on lung inflammation and tumor formation. Sham: sham-operated, OVX: ovariectomized, C: control, N+T: NNK+TCDD, N+E2: NNK+E2 (17β-estradiol), N+E2+T: NNK+E2+TCDD.

We next examined whether lung inflammation and proliferation could also be observed in lung tissues after a long-term experiment. We found that the expression of COX-2, iNOS, MMP-9, and PCNA were increased in groups treated with NNK combined with TCDD, E2, or both, in which E2 had strong effects on promoting NNK-induced inflammation and proliferation in the long-term exposure experiment (Figure 5a). Similar to the above results, co-administration of TCDD in the NNK+E2 group rendered the inflammation and proliferation effects of NNK combined with E2. The results suggested that A/J mice given TCDD or E2 over a long-term period can potentiate the NNK-induced activation of MAPK signaling that mediates an increase in inflammation and cell proliferation, leading to lung tumorigenesis. E2 combined with NNK showed the most potent lung tumorigenic effects among all treatment groups (Figure 5b).

## Discussion

NNK and TCDD are both well-known human carcinogens that have been proved to induce lung cancer formation in animal studies. Our previous study indicated that TCDD promoted NNK-induced lung tumor in female A/J mice, implying that estrogen and environmental toxicants such as TCDD, NNK could cooperate the mechanisms of lung tumorigenesis [6]. It has been reported that chronic inflammation induced by environmental toxicants is an important mechanism in lung tumorigenesis [16]. Therefore, the short-term exposure model in the present study was performed to identify whether NNK combined with TCDD or E2 induced inflammation. To delineate the relationship between inflammation and lung tumorigenesis, we performed the long term exposure model. Tumor promoting effects of E2 and TCDD when combined with NNK was also investigated. Moreover, the possible interaction in mice exposed to NNK in combination with TCDD, E2, or both, in inducing lung tumorigenesis was investigated. Figure 5b proposes the mechanistic model, showing that E2 possesses a strong enhancing effect on NNK-induced lung inflammation through activation of the MAPK and NFκB pathways, leading to COX-2 overexpression. This inflammatory response could be correlated with the potentiated effects of E2 on lung tumor promotion. On the contrary, co-administration of TCDD with NNK and E2 decreased inflammation, tumor incidence and multiplicity when compared with NNK+E2. Our current findings suggest that NNK and E2 together significantly induce lung tumor formation in OVX mice, implying that the induction of lung tumor formation in intact NNK-treated mice is hormone-related. Besides, TCDD may antagonize lung tumorigenic effect induced by E2.

In this report, we used a murine model of lung adenocarcinoma induced by NNK, and have demonstrated the lung tumor promoting effects of E2 was more potent than TCDD. We have shown that the tumor multiplicity and the inflammatory response were more obviously by E2 treatment, indicating that E2 can strongly potentiate inflammation and tumorigenesis induced by NNK (Table 2). These results are consistent with previous studies showed that estrogen not only promotes the malignant development of breast cancer, but also acts on non-target organs, including lung cancer through various molecular mechanisms [21], [22]. In addition to the solitary effect of estrogen, females exposed to other environmental chemicals such as benzo[a]pyrene that may interfere with the physiological functions of estrogen leading to increasing susceptibility of cancers [23]–[25]. However, our present study indicated that NNK combined with E2 and TCDD did not show an addictive effect in lung tumor formation, instead, the tumor multiplicity of NNK+TCDD+E2 groups was lower than NNK+E2 groups (Table 2). Those results are consistent with others, suggesting that TCDD exposure antagonize a wide spectrum of estrogen induced responses, including cell proliferation [26]. A number of mechanisms have been suggested, including that TCDD down regulation of the estrogen receptor (ER) in the uterus and other organs by activation of aryl hydrocarbon receptor (AhR) [27]. Mechanistic study indicated that TCDD up-regulates estrogen metabolizing enzymes, such as the CYP1 family, to enhance the metabolism of estradiol to inactive metabolites, thereby decreasing the half-life of active estrogen [28]. TCDD induced inhibition of estrogen-stimulated growth in MCF-7 cells could be due to the block entry into S phase by inhibiting the activation of cdk2, cdk4, and Rb [29]. Those above findings can provide evidence, at least in part, to support our finding that TCDD antagonized the lung tumor promoting effects of estrogen.

The precise mechanisms by which estrogens or TCDD influence the lung tumorigenesis is unknown. One recent study indicated that the induction of an NFκB-dependent inflammatory response in lower airways after prolonged exposure to cigarette smoke could be involved in lung tumorigenesis in animals [30]. Our previous study and others showed that cigarette smoke modulate the expression of genes involved in estrogen metabolism that increases accumulation of 2OHE2 and 4OHE2 in lung cells, and lead to NFκB activation, COX-2 expression, and lung carcinogenesis [16], [31]. Thus, we examined inflammatory response in the lung tissue and revealed that NNK combined with E2, TCDD or both significantly increased translocation of NFκB subunits (p65 and p50) from cytoplasm to nucleus, evidenced by the findings that the expression of cytoplasmic p65 and p50 were decreased, while nuclear p65 and p50 were increased (Figure 3b). Meanwhile, the expression of COX-2, which is an NFκB downstream inflammatory mediator, was increased. Interestingly, NNK+E2 groups had strengthened the expression of these inflammatory proteins (Figure 3). The results were consistent with tumor incidence and multiplicity observed in the long-term study that E2 had potent lung tumorigenic effect when combined with NNK. Although the mechanisms of inflammatory induced by E2 or TCDD are not well understood, continuous to inflammation is reported to increase lung cancer development. Thus, a continuous inflammatory response driven by E2 or TCDD could potentiate lung tumorigenesis by NNK (Figure 5). However, TCDD co-administered with NNK+E2 induces less lung tumor formation as well as inflammation responses compared to NNK+E2 group (Figure 3 and Table 2). As reported earlier, TCDD may interact with NFκB pathway through AhR-mediated signaling that AhR and NFκB physically interact, thereby causing mutual repression [32]–[34]. Nevertheless, our results showed that NNK combined with TCDD did not inhibit the expression of inflammatory-related proteins (Figure 3); thus, it is possible that TCDD co-administrated to NNK+E2 induced less inflammation compared to NNK+E2 could be due to the antagonistic effect of TCDD and E2.

MAPK pathway (includes ERK1/2, JNK, and p38 kinases) is the most extensively investigated intracellular signaling cascade involved in NFκB activation and the pro-inflammatory [35]. Our results further implied that NNK combined with E2 appeared prone to activate MAPK pathways (Figure 4). In agreement with our findings, Majidi *et al.* reported that E2 significantly enhances cell proliferation in response to NNK through NFκB and MAPK signaling pathways. The report suggested cooperation between NNK-induced β-AR and β-ER mitogenic signaling, leading to the up-regulation of the Ras/Raf/ERK1/2Elk1 signaling pathway in lung epithelial cells [36]. Upon activation of MAPK signaling pathways, various pro-inflammatory cytokines including TGF-β, TNF-α, IL-6, and IL-8 were released. Our present results indicate that TGF-β production increases in the short-term experiment then declined, whereas, in the long- term experiment, TNF-α significantly increases after treatment with NNK and in the combined treatment groups (Figure 3a). Consistently, Biseli *et al.* indicated that TGF-β levels were increased in the lung airway and lung parenchyma after a 2-month exposure to cigarette smoke condensates(CSC) and may be the stimulus responsible for collagen fiber deposition in lung parenchyma [37], [38]. Another study indicated that TGF-β increased after a single CSC exposure for 2 h then declined over time after 6 months of CSC exposure. The apparent down-regulation of TGF-β expression with long-term exposure may be more complex, TGF-β may switched on and off in different cell populations at different times [39]. Loss of the TGF-β response has been shown to be associated with tumor development and/or tumor progression in some cancer cell lines [40], [41]. Therefore, these reports support our finding that TGF-β increases in response to the short-term exposure model that may be involved in regulating the inflammatory response; however, the decline in TGF-β in the long-term exposure model may be correlated with tumor progression or metastasis.

An unexpecting finding of our experiments was that the expression of TNF-α increased in the long-term exposure model but not in the short-term model. One recent study also found that TNF-α release was increased after a 3 month exposure to cigarette smoke in lung tissues [42]. The molecular mechanisms of TNF-α in tumor progression are due to its ability to activate multiple signaling pathways, including NFκB, that play critical roles in mediating cell proliferation and survival [43]. Therefore, Karabela *et al.* reported that TNF-α neutralization is effective against urethane-induced lung oncogenesis in mice [44]. These reports indicated that increased TNF-α may contribute to inflammation-associated tumorigenesis.

Current knowledge gives new insight into the etiology of lung cancer development is a complex pathway mediated not by a single carcinogen, but rather by the synergistic interaction between environmental chemicals, hormones and cigarette smoke carcinogens [4]. This interaction raises public health concerns and challenging questions [1]. Until now, to delineate the complex relationship between exposure to multiple hazardous chemicals and lung carcinogenesis is still difficult. The present study is the first to explore the differential mechanisms of combined exposure by both short-term and long-term exposure animal models. This finding is of particular important to women as they are at risk for lung cancer development due to cigarette smoking exposure. It is appears that E2 stimulates NNK-induced activation of MAPK signaling, which correlates with increase in inflammation, cell proliferation, and tumorigenesis. Furthermore, NNK may increase the accumulation of carcinogenic E2 metabolite 4-OHEs and decreases the protective 2-OMeE species in lung tissues [45]. However, TCDD co-administered with NNK+E2 may antagonize tumor promoting effect by E2. Further studies are needed to determine the exact mechanisms of how E2 promote NNK-induced lung cancer and the contradictory mechanisms of TCDD and E2.
